# Flat building blocks for flat silicene

**DOI:** 10.1038/s41598-017-11360-4

**Published:** 2017-09-07

**Authors:** Masae Takahashi

**Affiliations:** Graduate School of Agricultural Science, Tohoku University, Sendai, 980-0845 Japan

## Abstract

Silicene is the silicon equivalent of graphene, which is composed of a honeycomb carbon structure with one atom thickness and has attractive characteristics of a perfect two-dimensional π-conjugated sheet. However, unlike flat and highly stable graphene, silicene is relatively sticky and thus unstable due to its puckered or crinkled structure. Flatness is important for stability, and to obtain perfect π-conjugation, electron-donating atoms and molecules should not interact with the π electrons. The structural differences between silicene and graphene result from the differences in their building blocks, flat benzene and chair-form hexasilabenzene. It is crucial to design flat building blocks for silicene with no interactions between the electron donor and π-orbitals. Here, we report the successful design of such building blocks with the aid of density functional theory calculations. Our fundamental concept is to attach substituents that have *sp*-hybrid orbitals and act as electron donors in a manner that it does not interact with the π orbitals. The honeycomb silicon molecule with BeH at the edge designed according to our concept, clearly shows the same structural, charge distribution and molecular orbital characteristics as the corresponding carbon-based molecule.

## Introduction

**Figure 1:**
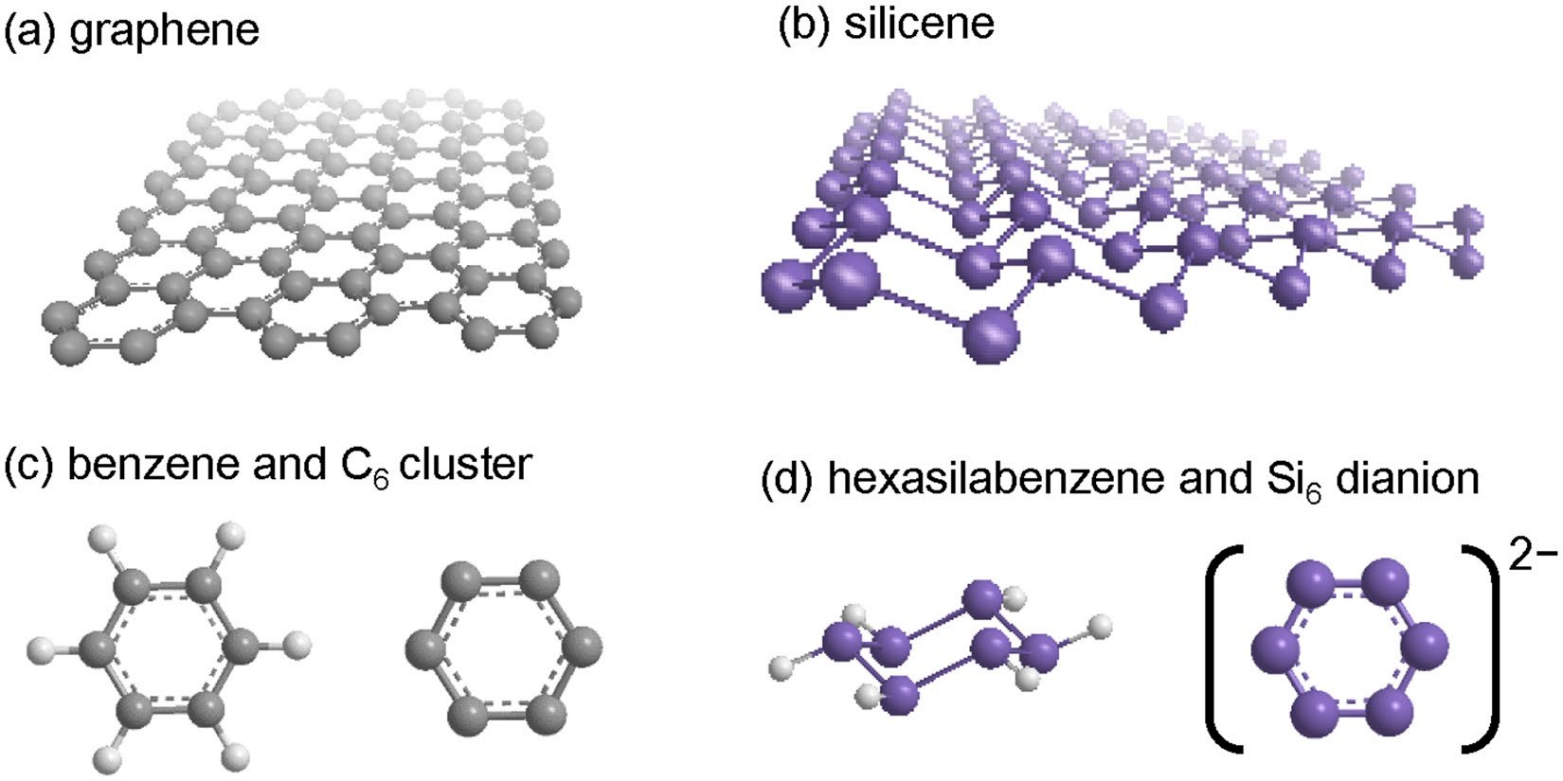
2D sheets of carbon and silicon and their building blocks. Graphene (**a**), silicene (**b**), benzene and a C_6_ cluster (**c**), and hexasilabenzene and a Si_6_ dianion cluster (**d**).

Silicene is the silicon equivalent of graphene, which is a famous material composed of a honeycomb layer of carbon just one atom thick^1–3^. If applied to electronic devices, these silicene layers would enable the semiconductor industry to achieve ultimate miniaturization^4, 5^. However, unlike flat and highly stable graphene (Fig. 1a), silicene is relatively sticky and unstable due to its puckered or crinkled structure (Fig. 1b)^6^. To produce silicene, the hot vapor of silicon atoms is condensed onto crystalline blocks of silver^7–16^ or other materials^17–19^. However, the substrate can affect the π conjugated system. Recent twisted silicene multilayers have proved the two-dimensional (2D) nature of silicene and was a major progress toward flat silicene^20^, but interlayer interactions still perturb the electronic structure of 2D materials.

The building blocks of graphene are benzene and/or C_6_ clusters (Fig. 1c), both of which are flat^21, 22^. In contrast, hexasilabenzene, which is a building block of silicene, is not flat but instead has a chair form (Fig. 1d)^23^. Benzene is an archetypical aromatic hexagon composed of carbon with delocalized π electrons. The 2D aromaticity of flat monocyclic systems is governed by the Hückel 4 *N* + 2 rule, where N is the number of π electrons. The outstanding properties of C_6_ clusters include double aromaticity^24^ with an orthogonal Hückel framework (an out-of-plane p_π_ orbital and an in-plane radial-type orbital contributing to σ-aromaticity in a single molecule). We previously discovered a similar flat silicon hexagon with double aromaticity, i.e., the dianion of the Si_6_ cluster (Fig. 1d)^25, 26^. This finding encouraged us to design the present flat silicene building blocks using electron-donating substituents. A pioneering work recently reported the formation of flat silicene via doping with calcium as an electron donor^27^, and attempts to make flat hexasilabenzene and Si_6_ clusters were carried out earlier with Zintl anions^28, 29^. In either case, the Si_6_ hexagon is surrounded by an electron donor, creating a three-dimensional crystal but not a pure 2D crystal.

To realize a perfect and stable π-conjugated 2D sheet of silicene, the sheet must be flat and have no electron-donating atoms or molecules interacting with the π electrons. The only way to donate electrons without disturbing the out-of-plane π orbital is to modify the in-plane edges. A theoretical study of silicene molecules (large polycyclic molecules consisting of six-membered silicon rings) from Si_13_H_9_ to Si_60_H_24_ showed that silicene molecules with hydrogen atoms at the edge are not flat but have a low-buckled structure^30^. This indicates that, as described above, electron-donating substituents are good candidates for the formation of flat silicene molecules and hydrogen atoms are not suitable. Although a theoretical attempt to use an electron-donating metal, such as lithium, at the in-plane edge gave the *D*_*6h*_ planar structure of hexasilabenzene^31–33^, the lithium atoms were not attached to one silicon atom but instead attached between two adjacent silicon atoms due to the ability of silicon to form three-center bonds with lithium. Furthermore, the bicyclic chain is no longer flat when lithium is used as the electron donor but is puckered with a zigzag chain. A completely different concept is necessary for the design of a flat polycyclic molecule consisting of silicon hexagons as flat silicene building blocks. Here, we report the successful design of flat 2D molecules composed of six-membered silicon rings with modifications at the in-plane edges using density functional theory (DFT) calculations.

## Results

### Basic concept

**Figure 2:**
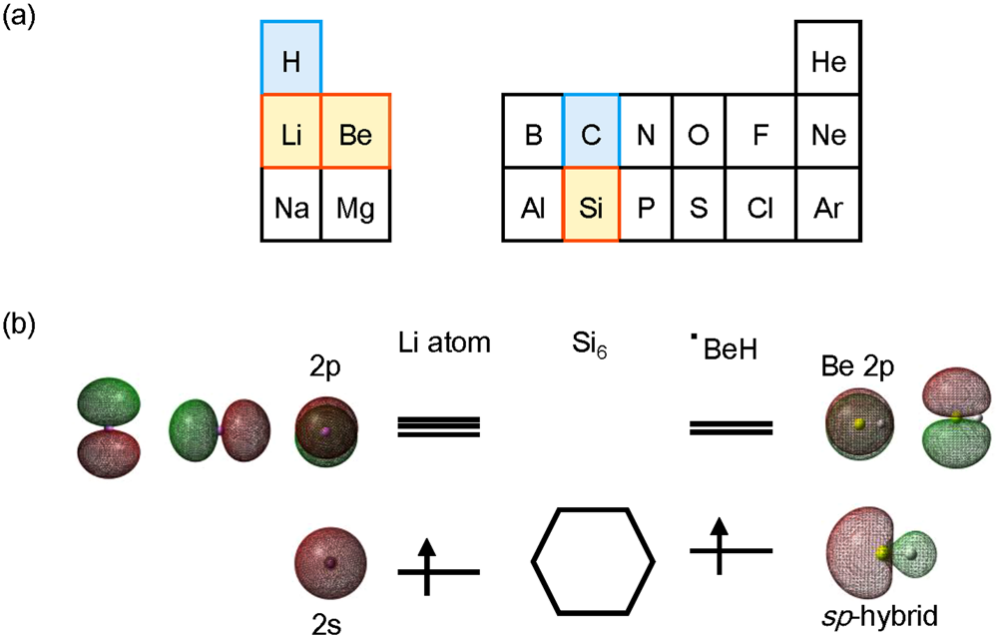
(**a**) The periodic table of elements H–Ar. (**b**) The molecular orbitals of an Li atom and BeH radical. The orbitals were calculated at the B3LYP/cc-pVTZ level and are depicted at the 0.02 isovalue.

**Figure 3:**
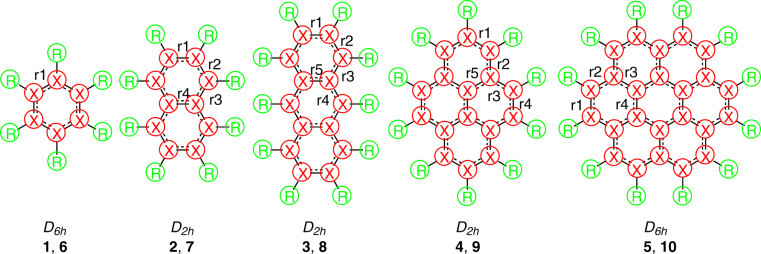
Calculated molecules. **1**–**5**: X = Si, R = BeH; **6**–**10**: X = C, R = H. The notation r1–r5 represents the bond length between the adjacent X atoms.

Our basic concept for the design of flat polycyclic molecules consisting of silicon hexagons is to use *sp*-hybrid orbitals (Fig. 2b) with electron-donating ability. We first considered the differences between C-H and Si-H interactions. Notably, the energies of the interacting orbitals are different. That is, the energy difference between the H 1s and Si 3p orbital is larger than that between the H 1s and C 2p orbital. The use of a lithium 2s orbital instead of a hydrogen 1s orbital is a straightforward way to compensate for the energy difference (Fig. 2a). However, unlike hydrogen, the empty 2p orbital (Fig. 2b) of lithium interacts with the silicon 3p orbital and forms a three-center bond with two adjacent silicon atoms of the silicon ring. Eventually, we decided to use linear *sp*-hybrid orbitals and selected BeH as the substituent. First-year chemistry textbooks state that BeH_2_ has a linear shape due to its *sp*-hybrid orbital. Figure 2b shows the molecular orbital of an Li atom and BeH radical for comparison. The difference between the two singly occupied molecular orbitals (SOMOs) is that the SOMO of the BeH radical points to the Si_6_ ring (Fig. 2b). Silicon equivalents **1**, **2**, **3**, **4** and **5** of benzene (**6**), naphthalene (**7**), anthracene (**8**), pyrene (**9**) and coronene (**10**), respectively, were selected as building blocks and substituted with BeH at the ring edge (Fig. 3).

### Optimized structure

The five silicon-based molecules **1**–**5** with BeH at the ring edge show the same structural, charge distribution and molecular orbital features as the corresponding carbon-based molecules **6**–**10**, where BeH is used according to the above concept. All of the calculated molecules **1**–**10** have a flat structure as their minimum with no imaginary frequencies. Electron donation stabilizes the flat structure of the six-membered silicon ring and further provides the stable flat structure of polycyclic silicon molecules. The lowest vibrational frequency modes (*ν*_*1*_), given in Table 1, are the out-of-plane motion of the terminal BeH moiety (**3**) and the out-of-plane ring deformation (**1**, **2**, **4**–**10**), respectively. The calculated silicon-silicon bond length (2.22 to 2.28 Å) is between the silicon-silicon single and double bond length (the bond lengths of H_3_Si–SiH_3_ and H_2_Si = SiH_2_ at the B3LYP/cc-pVTZ level are 2.355 Å and 2.166 Å, respectively), which is similar to the structure of aromatic benzene having a bond length between the carbon-carbon single and double bond. The silicon-silicon bond lengths are equal in hexasilabenzene **1** due to its *D*_*6h*_ symmetry but differ at most by 0.06 Å in the other molecules **2**–**5**. The carbon-based structures **6**–**10** show the same behavior. Benzene **6** has the same carbon-carbon bond lengths throughout the ring, while the others **7**–**10** have slightly different bond lengths, with the difference being at most 0.08 Å. Notably, the terminal BeH is not located between the two silicon atoms in the minimized structures of **1**–**5**, which is markedly different from the lithium-terminated six-membered ring Si_6_Li_6_^31^.

**Table 1:** Optimized geometry, charge (Q_ring_), frequency (*ν*_*1*_) and HOMO-LUMO gap of molecules **1**–**10** at the B3LYP/cc-pVTZ level.

Molecule	Bond length^a^/Å	Q_ring_^b^	*ν*_*1*_^c^ */*cm^−1^	H-L gap^d^/eV
**1**	2.252/r1	−2.2	47.57	1.90 (2.60)
**2**	2.267/r1, 2.234/r2, 2.267/r3, 2.266/r4	−3.0	22.22	1.61 (1.94)
**3**	2.274/r1, 2.229/r2, 2.274/r3, 2.250/r4, 2.274/r5	−3.8	13.41	1.21 (1.51)
**4**	2.248/r1, 2.252/r2, 2.280/r3, 2.222/r4, 2.263/r5	−3.8	15.26	1.39 (1.58)
**5**	2.231/r1, 2.268/r2, 2.255/r3, 2.263/r4	−4.6	14.80	1.24(1.71)
**6**	1.391/r1	−1.2	413.94	5.47 (6.70)
**7**	1.412/r1, 1.370/r2, 1.416/r3, 1.428/r4	−1.6	173.54	4.39 (4.79)
**8**	1.421/r1, 1.363/r2, 1.425/r3, 1.395/r4, 1.440/r5	−2.0	91.43	3.22 (3.57)
**9**	1.388/r1, 1.399/r2, 1.433/r3, 1.355/r4, 1.423/r5	−2.0	99.20	3.68 (3.84)
**10**	1.366/r1, 1.419/r2, 1.416/r3, 1.423/r4	−2.4	88.27	3.22 (4.03)

^a^The notation r1−r5 represents the bond length between the X atoms (X = Si, C) shown in Fig. 3. ^b^Q_ring_: Summation of the NPA charges on the ring. ^c^ *ν*_*1*_: The lowest vibrational frequency. ^d^HOMO-LUMO gap from the first excitation energies obtained by TDDFT/B3LYP. HOMO-LUMO gap from the Kohn-Sham eigenvalues of the ground state DFT/B3LYP calculation is given in parentheses.

### Charge distribution

**Figure 4:**
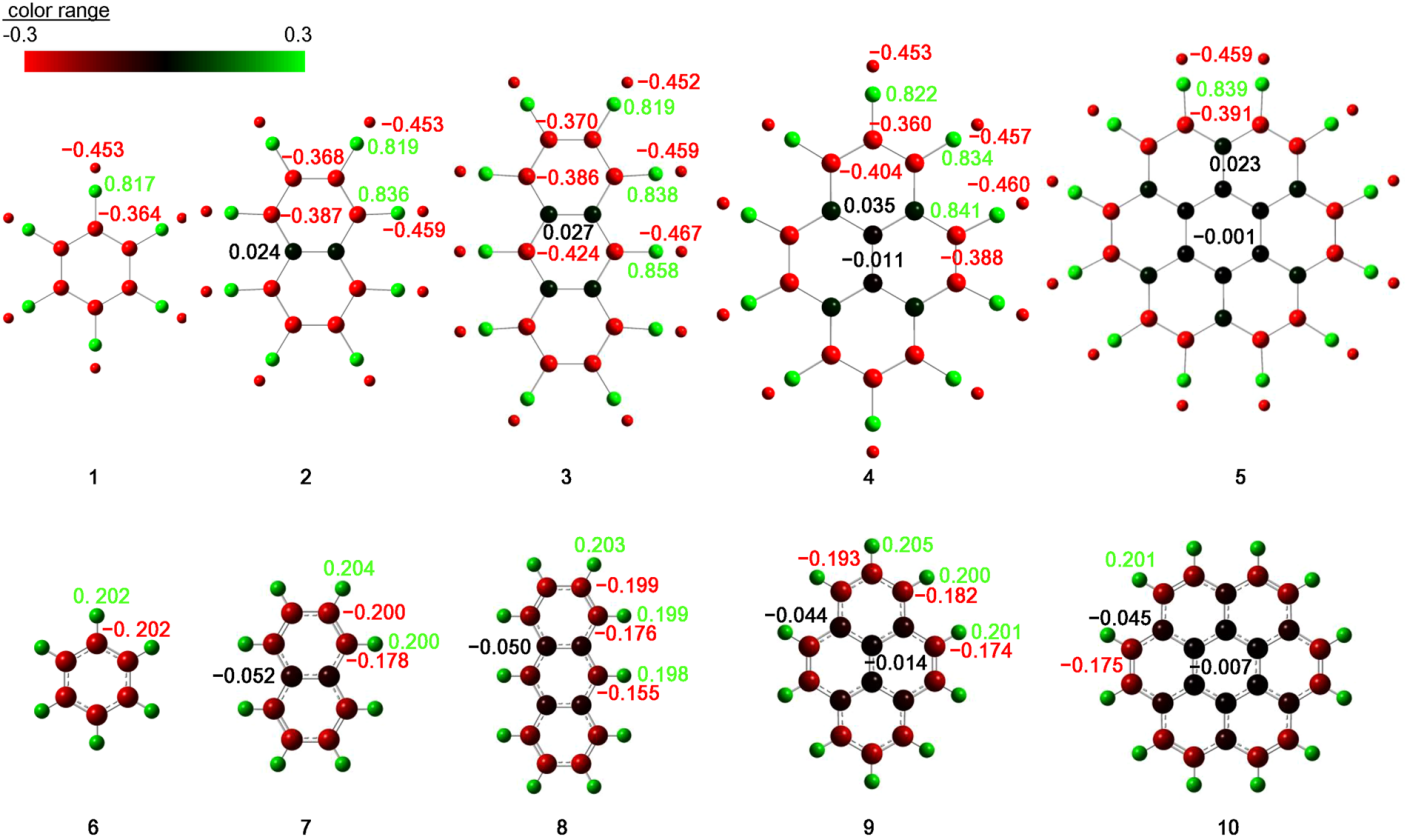
Charge distribution of molecules **1**–**10**. Each atom is colored according to the charge, and the number indicates the charge.

As shown in Table 1, the total NPA charges on the silicon rings (Q_ring_) of **1**–**5** are all negative, indicating electron donation from the terminal BeH to the ring moiety. Figure 4 compares the charge distribution in molecules **1**–**5** to that of **6**–**10**. The charge of the ring-edge silicon, at which BeH is directly bound, is strongly negative (red), while that of the silicon inside the ring is nearly neutral (black). The charge of the Be atom is strongly positive (green), while that of the hydrogen in BeH is strongly negative (red). These trends are the same as those in **6**–**10**, but the contrast between the negative and positive charges is slightly enhanced in the silicon-based molecules **1**–**5**. The total NPA charge on the carbon ring (Q_ring_) of **6**–**10** is negative, and the ring is surrounded by positively charged hydrogen atoms. The edge carbon is negatively charged, and the inside of the ring is nearly neutral. The neutrality of the charge inside the ring is more evident in larger molecules. The charge distribution indicates that BeH terminates the silicon equivalents **1**–**5** as well as the hydrogen atoms of **6**–**10**.

### Molecular orbitals

**Figure 5:**
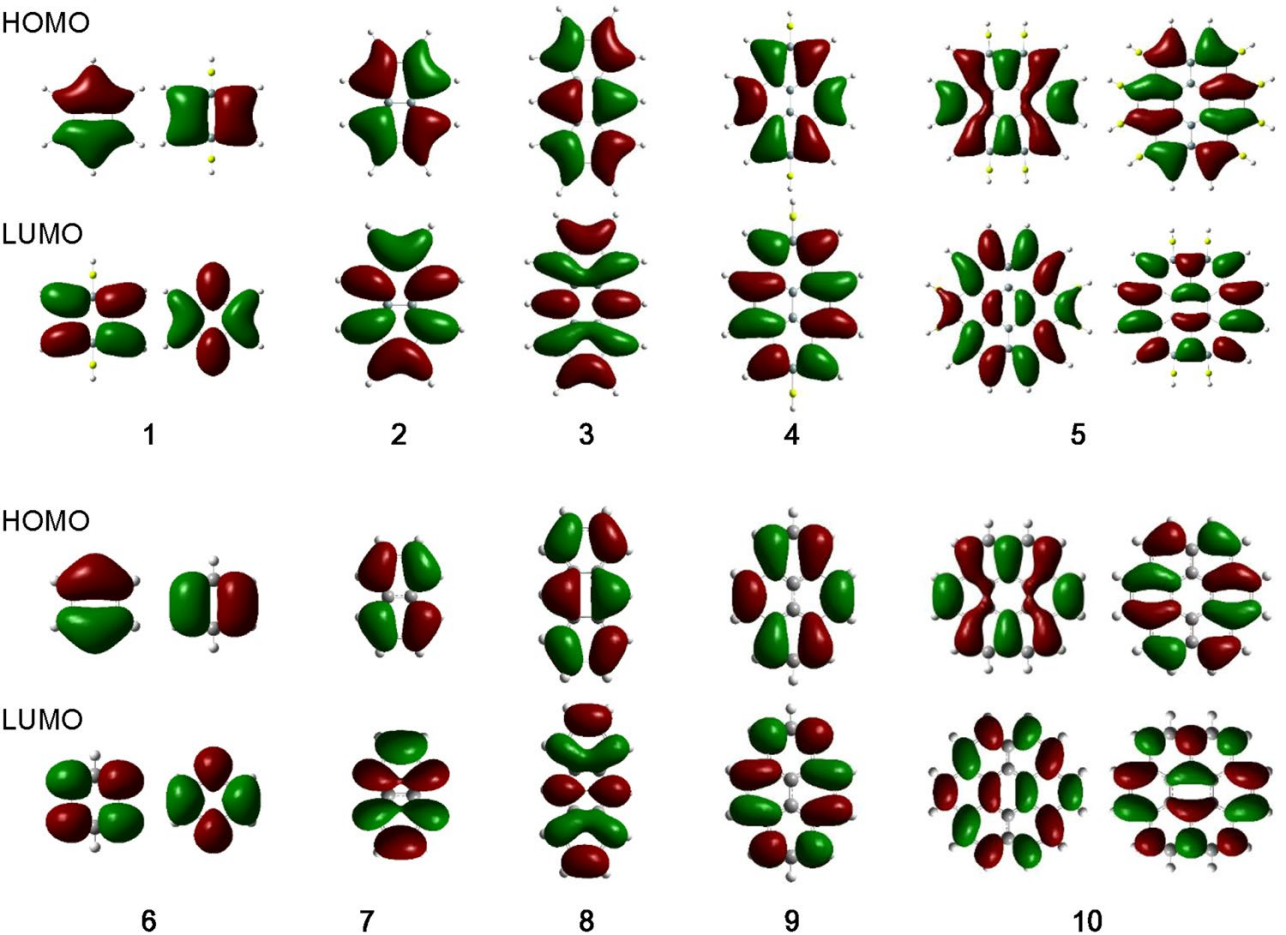
HOMO and LUMO of molecules **1**–**10**. The molecular orbitals were calculated at the B3LYP/cc-pVTZ level and are depicted at the 0.01 and 002 isovalues for **1**–**5** and **6**–**10**, respectively.

Pure π-conjugation is a key property of flat silicene as well as flat graphene. The highest occupied molecular orbitals (HOMOs) and the lowest unoccupied molecular orbitals (LUMOs) are shown in Fig. 5. The HOMO and LUMO of **1**–**5** are all confirmed to be π orbitals. Their shapes are similar to the HOMO and LUMO of **6**–**10**, but slightly delocalized on the Be atoms. Thus our design using a linear *sp*-hybrid orbital at the terminus is expected to successfully provide the building blocks for flat and stable π-conjugated silicene. The HOMO-LUMO gap of all the proposed precursor molecules are listed in Table 1.

### Other hexagons and isomers

**Figure 6:**
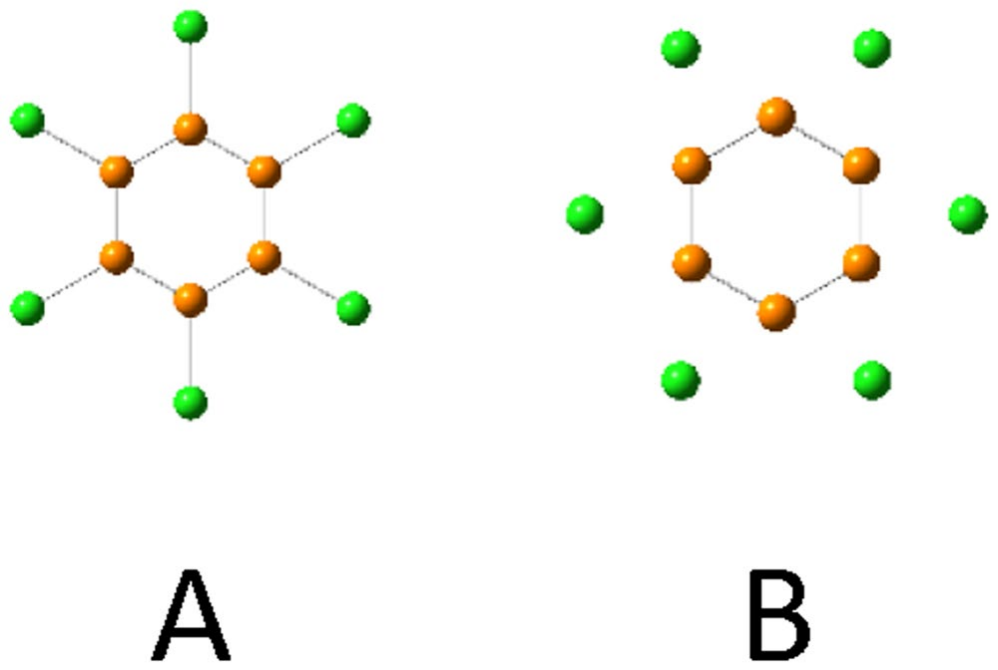
Two *D*_*6h*_ structures. A: benzene-like, B: Si_6_Li_6_-like where metal moves between two silicon atoms.

Several other hexagons X_6_R_6_ (X = Si, Ge) were calculated to investigate which R substituents, other than Si_6_(BeH)_6_, can be used for the design of flat hexagons (Table 2). Since our aim is to obtain a six-membered ring with *D*_*6h*_ symmetry, we optimized the structure under the constraint of *D*_*6h*_ symmetry. In case of metal substitution at ring edge, two types of structures have the symmetry of *D*_*6h*_ (Fig. 6): benzene-like (A) and Si_6_Li_6_-like where metal moves between two silicon atoms (B). For comparison, the results of Si_6_H_6_ and Si_6_Li_6_ of structure A at the same level of calculations as our present work are also listed in Table 2. As is well known, our results also showed benzene-like structure of Si_6_H_6_ and Si_6_Li_6_ did not give a minimum.

**Table 2:** Optimized hexagonsX _6_R_6_ with *D*_*6h*_ symmetry.

X	R	Stationary point^a^	Imaginary Mode of 1^st^ TS	Structure^b^
Si	Na	3^rd^ TS		B
Si	K	1^st^ TS	Our-of-plane motion of K	B
Si	Mg	2^nd^ TS		B
Si	Ca	6^th^ TS		B
Si	Cu	1^st^ TS	Out-of-plane ring deformation	A
Si	Zn	1^st^ TS	Our-of-plane motion of Zn	B
Si	C≡N	1^st^ TS	Out-of-plane ring deformation	A
Si	MgH	MIN		A
Si	CaH	6^th^ TS		A
Ge	BeH	MIN		A
Ge	MgH	1^st^ TS	Our-of-plane motion of MgH	A
Ge	CaH	17^th^ TS		A
Si	H	1^st^ TS	Out-of-plane ring deformation	A
Si	Li	6^th^ TS		A

Full geometry optimizations were performed at the B3LYP/6-311++ G(3df,3pd) level for K, Ca, Cu, Zn and CaH substitution, and at the B3LYP/cc-pVTZ level for H, Li, Na, Mg, C≡N, BeH and MgH substitution. ^a^MIN: minimum, TS: transition state. ^b^Two *D*_*6h*_ structures A and B are shown in Fig. 6. More stable structure between the two *D*_*6h*_ structures is listed for the terminal substituent of metals (R = Na, K, Mg, Ca, Cu, Zn). For comparison, the results of Si_6_H_6_ and Si_6_Li_6_ of structure A are listed.

Among the optimized hexagons in Table 2, a flat *D*_*6h*_ ring was obtained as the minimum for only the MgH-terminated six-membered silicon ring and the BeH-terminated six-membered germanium ring. In both hexagons, MgH and BeH have an *sp*-hybrid orbital and acts as an electron donor. In the optimized structure, BeH and MgH do not migrate to between the silicon atoms or germanium atoms. The optimized silicon-silicon and germanium-germanium bond lengths are 2.264 Å and 2.350 Å, respectively. Although the minimized geometry of the MgH-terminated six-membered silicon ring has a flat structure, the planar hexagon is not the minimum of the MgH-terminated six-membered germanium ring but is instead its 1^st^ order transition state (TS). Furthermore, with CaH substitution, both the six-membered silicon and germanium rings become considerably higher order TS. Therefore, light metals are better for the design of flat hexagons. The imaginary mode of MgH-terminated six-membered germanium ring is the out-of-plane motion of MgH, which leads to a nonplanar structure. This motion does not cause MgH to migrate between two germanium atoms. The lowest vibrational frequency of BeH-terminated six-membered germanium ring is 22.24 cm^−1^, which is much smaller than that of BeH-terminated six-membered silicon ring (47.57 cm^−1^). This makes it difficult to design flat building blocks for flat germanene. A proper substituent at the ring edge is required to tune the charge on the ring and the bond strength between Ge and the substituent.

Several monovalent and divalent metals with electron-donating ability were examined as substituents (R = Na, K, Mg, Ca, Cu, Zn), but these did not produce flat *D*_*6h*_ hexagons as the minimum. Table 2 lists more stable structure between the two *D*_*6h*_ structures, benzene-like (A) and Si_6_Li_6_-like (B). Three are 1^st^ order TSs, and the others are higher order TSs. The imaginary modes of the 1^st^ order TSs are the out-of-plane motion of the metal (R = K and Zn) and out-of-plane ring deformation (R = Cu), leading to a nonplanar minimum. As mentioned before, the lithium-terminated six-membered silicon ring has a *D*_*6h*_ planar structure^31–33^, and the lithium atom is not attached to one silicon but between two adjacent silicon atoms. Cu showed a benzene-like structure (A), while the other metals migrated between two silicon atoms (B) similar to lithium. Thus, simple electron donation is insufficient for the design of flat hexagons. Next, for the substituent with an *sp*-hybrid orbital (R = (C≡N)), the *D*_*6h*_ structure was obtained as the 1^st^ order TS. The imaginary mode is out-of-plane ring deformation, leading to a nonplanar minimum. Therefore, a simple *sp*-hybrid orbital without electron donation is also insufficient for the design of flat hexagons.

Hexasilabenzene is known to be less stable than many of its isomers. For example, synthetically accessible hexasilaprismane^34–36^ is more energetically stable. To examine the relative stability between the isomers of our system, we calculated the six valence isomers^37, 38^ of Si_6_(BeH)_6_ and obtained the silicon equivalent of benzvalene^39^, prizmane^40^ and bicyclopropenyl^41^ in addition to benzene (**1**) as a minimum. Among the four, BeH-terminated hexasilabenzvalene is the most stable at the B3LYP/cc-pVTZ level of calculation. Since BeH-terminated hexasilabenzene is not the most stable isomer, the step-by-step manufacture of flat silicene from BeH-terminated hexasilabenzene is not recommended. It would be better to prepare zigzag silicene and then terminate it with BeH.

## Discussion

Here, we designed flat building blocks **1**–**5** to construct flat silicene using DFT calculations. We used substituents with *sp*-hybrid orbitals that act as electron donors. The minimum structure of all the obtained silicon polycyclic molecules **1**–**5** is flat. The charge is nearly neutral inside the ring and strongly negative at the ring edge due to the terminal BeH substituent. The HOMO and LUMO of **1**–**5** are π-orbitals. The designed molecules **1**–**5** could act as building blocks for flat silicene, which is a π-conjugated 2D sheet composed of six-membered silicon rings. The difference between the silicene constructed here and graphene is that BeH must be present at the terminal to stabilize the flat structure. In this sense, the molecules presented here are building blocks of silicene ribbons due to the existence of a terminal substituent. Flat six-membered silicon rings have long been desired in silicon chemistry and 2D silicon materials. In this study, flat hexasilabenzene was realized, and it was confirmed that the extended ring molecules are also flat. The flatness of these building blocks opens the way to flat silicene ribbons or films constructed by them, but at present it does not guarantee the flatness of silicene ribbons or films. Further theoretical and experimental studies with careful design of the edge structure are required to realize flat silicene.

## Methods

### DFT calculations

DFT calculations were performed with the Gaussian 09 software package^42^. We utilized a hybrid Becke-type three-parameter exchange functional^43^ paired with the gradient-corrected Lee, Yang, and Parr correlation functional (B3LYP)^44, 45^ and the cc-pVTZ basis set^46–50^ unless otherwise noted. The geometric parameters were fully optimized, and the minimized structures were confirmed to have no imaginary frequencies. To investigate the charge at each atom, natural population analysis (NPA)^51–56^ charges were calculated, as NPA charges are less basis-set dependent. Time-dependent density functional theory (TDDFT) calculations were carried out to obtain the HOMO-LUMO gap using the optimized ground state geometries^57^. In all molecules studied here, the first excitation is mainly contributed by the HOMO-LUMO transition, although some of them are forbidden with zero oscillator strength. In Table 1, we list the first excitation energies as the HOMO-LUMO gap.

## References

[1] Guzmán-Verri GG, Voon LCLY (2007). Electronic structure of silicon-based nanostructures. Phys. Rev. B.

[2] Voon LCLY, Zhu J, Schwingenschlögl U (2016). Silicene: Recent theoretical advances. Appl. Phys. Rev.

[3] Zhao J (2016). Rise of silicene: A competitive 2D material. Prog. Mater. Sci..

[4] Tao L (2015). Silicene field-effect transistors operating at room temperature. Nature Nanotechnol.

[5] Peplow M (2015). Silicene makes its transistor debut. Nature.

[6] Brumfiel G (2013). Sticky problem snares wonder material. Nature.

[7] Vogt P (2012). Silicene: Compelling experimental evidence for graphenelike two-dimensional silicon. Phys. Rev. Lett..

[8] Feng B (2012). Evidence of silicene in honeycomb structures of silicon on Ag(111). Nano Lett..

[9] Gao J, Zhao J (2012). Initial geometries, interaction mechanism and high stability of silicene on Ag(111) surface. Sci. Rep.

[10] Kaltsas D, Tsetseris L, Dimoulas A (2012). Structural evolution of single-layer films during deposition of silicon on silver: A first-principles study. J. Phys.: Condens. Matter.

[11] Guo Z-X, Furuya S, Iwata J-I, Oshiyama A (2013). Absence and presence of Dirac electrons in silicene on substrates. Phys. Rev. B.

[12] Wang Y-P, Cheng H-P (2013). Absence of a Dirac cone in silicene on Ag(111): First-principles density functional calculations with a modified effective band structure technique. Phys. Rev. B.

[13] Tsoutsou D, Xenogiannopoulou E, Golias E, Tsipas P, Dimoulas A (2013). Evidence for hybrid surface metallic band in (4 × 4) silicene on Ag(111). Appl. Phys. Lett..

[14] Moras, P., Mentes, T. O., Sheverdyaeva, P. M., Locatelli, A. & Carbone, C. Coexistence of multiple silicene phases in silicon grown on Ag(111). *J. Phys.: Condens. Matt**er* **26**, 185001, doi:10.1088/0953-8984/26/18/185001 (2014). 10.1088/0953-8984/26/18/185001 24727950

[15] Feng Y (2016). Direct evidence of interaction-induced Dirac cones in a monolayer silicene/Ag(111) system. Proc. Natl. Acad. Sci.

[16] Du Y (2016). Quasi-freestanding epitaxial silicene on Ag(111) by oxygen intercalation. Sci. Adv..

[17] Fleurence A (2012). Experimental evidence for epitaxial silicene on diboride thin films. Phys. Rev. Lett..

[18] Meng L (2013). Buckled silicene formation on Ir(111). Nano Lett.

[19] Chiappe D (2014). Two-dimensional Si nanosheets with local hexagonal structure on a MoS_2_ surface. Adv. Mater..

[20] Li Z (2016). Observation of van HOVE singularities in twisted silicene multilayers. ACS Cent. Sci.

[21] Minkin, V. I., Glukhovtsev, M. N. & Simkin, B. Y. *Aromaticity and antiaromaticity* (Wiley & Sons, Inc., 1994).

[22] Schleyer PvR (2001). Introduction: Aromaticity. Chem. Rev..

[23] Nagase S, Teramae H, Kudo T (1987). Hexasilabenzene (Si_6_H_6_). Is the benzene-like *D*_*6h*_ structure stable?. J. Chem. Phys..

[24] Schleyer PvR, Jiao H, Glukhovtsev MN, Chandrasekhar J, Kraka E (1994). Double aromaticity in the 3,5-dehydrophenyl cation and in cyclo[6]carbon. J. Am. Chem. Soc..

[25] Takahashi M (2010). Polyanionic hexagons: X_6_^n–^ (X = Si, Ge). Symmetry.

[26] Takahashi, M. & Kawazoe, Y. Theoretical study on planar anionic polysilicon chains and cyclic Si_6_ anions with *D*_*6**h*_ symmetry. *Organometallics* **24**, 2433–2440, doi:10.1021/om050025c (2005).

[27] Noguchi E (2015). Direct observation of Dirac cone in multilayer silicene intercalation compound CaSi_2_. Adv. Mater..

[28] Schnering, H. G. *et al*. Hückel arenes with ten π electrons: Cyclic Zintl anions Si_6_^10−^ and Ge_6_^10−^, isosteric to P_6_^4−^ and As_6_^4−^. *Angew. Chem. Int. Ed. Engl.* **35**, 984–986, doi:10.1002/anie.199609841 (1996).

[29] Nesper, R., Currao, A. & Wengert, S. Nonaromatic planar Si_12_ ring system of approximate *D*_*6h*_ symmetry in Ca_7_Mg_7.5±δ_Si_14_. *Chem. Eur. J.* **4**, 2251–2257, doi:10.1002/(SICI)1521-3765(19981102)4 (1998).

[30] Mokkath JH, Schwingenshlögl U (2016). Tunable optical absorption in silicene molecules. J. Mater. Chem. C.

[31] Zdetsis AD (2007). Stabilization of flat aromatic Si_6_ rings analogous to benzene: *Ab initio* theoretical prediction. J. Chem. Phys..

[32] Santos JC, Fuentealba P (2007). Aromaticity and electronic structure of silabenzenes. Possible existence of a new cluster Si_6_Li_6_. Chem. Phys. Lett..

[33] Zdetsis AD, Fowler PW, Havenith RWA (2008). Aromaticity of planar Si_6_ rings in silicon–lithium clusters. Mol. Phys..

[34] Sax A, Janoschek R (1986). Si_6_H_6_: Is the aromatic structure the most stable one?. Angew. Chem. Int. Ed. Engl..

[35] Sax AF, Kalcher J, Janoschek R (1988). MC-SCF and CI calculations on four isomers of Si_6_H_6_. J. Comput. Chem..

[36] Sekiguchi A, Yatabe T, Kabuto C, Sakurai H (1993). The “missing” hexasilaprismane: Synthesis, X-ray analysis, and photochemical reactions. J. Am. Chem. Soc..

[37] Balaban AT (1966). Valence-isomerism of cyclopolyenes. Rev. Roum. Chim..

[38] Takahashi M, Kawazoe Y (2006). Ab initio quantum chemical investigation of several isomers of anionic Si_6_. Chem. Phys. Lett..

[39] Wilzbach KE, Ritscher JS, Kaplan L (1967). Benzvalene, the tricyclic valence isomer of Benzene. J. Am. Chem. Soc..

[40] Katz TJ, Acton N (1973). Synthesis of prismane. J. Am. Chem. Soc..

[41] Billups WE, Haley MM (1989). Bicycloprop-2-enyl (C_6_H_6_). Angew. Chem. Int. Ed. Engl..

[42] Gaussian 09, Revision D.01, Frisch, M. J. *et al*. Gaussian, Inc., Wallingford CT, 2013.

[43] Becke AD (1993). Density-functional thermochemistry. III. The role of exact exchange. J. Chem. Phys..

[44] Lee C, Yang W, Parr RG (1988). Development of the Colle-Salvetti correlation-energy formula into a functional of the electron density. Phys. Rev. B.

[45] Miehlich B, Savin A, Stoll H, Preuss H (1989). Results obtained with the correlation energy density functionals of Becke and Lee, Yang and Parr. Chem. Phys. Lett..

[46] Dunning TH (1989). Gaussian basis sets for use in correlated molecular calculations. I. The atoms boron through neon and hydrogen. J. Chem. Phys..

[47] Kendall RA, Dunning TH, Harrison RJ (1992). Electron affinities of the first-row atoms revisited. Systematic basis sets and wave functions. J. Chem. Phys.

[48] Woon DE, Dunning TH (1993). Gaussian basis sets for use in correlated molecular calculations. III. The atoms aluminum through argon. J. Chem. Phys..

[49] Peterson KA, Woon DE, Dunning TH (1994). Benchmark calculations with correlated molecular wave functions. IV. The classical barrier height of the H + H_2_ → H_2_ + H reaction. J. Chem. Phys..

[50] Wilson AK, Mourik T, Dunning TH (1996). Gaussian basis sets for use in correlated molecular calculations. VI. Sextuple zeta correlation consistent basis sets for boron through neon. J. Mol. Struct. (Theochem).

[51] Foster JP, Weinhold F (1980). Natural hybrid orbitals. J. Am. Chem. Soc..

[52] Reed AE, Weinhold F (1983). Natural bond orbital analysis of near-Hartree–Fock water dimer. J. Chem. Phys..

[53] Reed AE, Weinstock RB, Weinhold F (1985). Natural population analysis. J. Chem. Phys..

[54] Reed AE, Weinhold F (1985). Natural localized molecular orbitals. J. Chem. Phys..

[55] Reed AE, Curtiss LA, Weinhold F (1988). Intermolecular interactions from a natural bond orbital, donor-acceptor viewpoint. Chem. Rev..

[56] Carpenter JE, Weinhold F (1988). Analysis of the geometry of the hydroxymethyl radical by the “different hybrids for different spins” natural bond orbital procedure. J. Mol. Struct. (THEOCHEM).

[57] Marques, M. A. L., Maitra, N. T., Nogueira, F. M. S., Gross, E. K. U. & Rubio, A. *Fundamentals of time-dependent density functional theory* (Springer-Verlag, vol. 837, 2012).

